# Prognostic Features of Near-Infrared Spectroscopy Following Primary Radical Prostatectomy

**DOI:** 10.3390/cancers13236034

**Published:** 2021-11-30

**Authors:** Tijl Vermassen, Jonas Himpe, Renaat Coopman, Charles Van Praet, Nicolaas Lumen, Sylvie Rottey, Joris Delanghe

**Affiliations:** 1 Department of Medical Oncology, University Hospital Ghent, 9000 Ghent, Belgium; sylvie.rottey@ugent.be; 2 Department of Clinical Chemistry, Microbiology and Immunology, Ghent University, 9000 Ghent, Belgium; jonas.himpe@ugent.be (J.H.); joris.delanghe@ugent.be (J.D.); 3 Department of Plastic, Reconstructive and Aesthetic Surgery, University Hospital Ghent, 9000 Ghent, Belgium; renaat.coopman@uzgent.be; 4 Department of Urology, University Hospital Ghent, 9000 Ghent, Belgium; charles.vanpraet@uzgent.be (C.V.P.); nicolaas.lumen@uzgent.be (N.L.)

**Keywords:** prostate cancer, local treatment, prognosis, NIR, spectroscopy, biochemical recurrence

## Abstract

**Simple Summary:**

Up to 53% of patients with localized/locally advanced prostate cancer (PCa) will be confronted with biochemical recurrence (BCR) at some point after primary radical prostatectomy. No robust prognostic biomarker exists in this patient setting. The aim of this retrospective study was therefore to determine if near-infrared (NIR) spectroscopy, an easy-to-use technique, could be an asset as a prognostic biomarker for PCa patients after radical prostatectomy. In a cohort of 82 patients, we confirmed that changes in the NIR spectrum of PCa specimens, obtained via radical prostatectomy, can be prognostic for BCR-free survival. The clinical implementation of NIR spectroscopy can therefore be a great asset as it can aid in the management of PCa patients following primary radical prostatectomy. Further elaboration is recommended.

**Abstract:**

Background: Only a few biomarkers have been evaluated for their prognostic value with regard to biochemical recurrence (BCR) following primary radical prostatectomy. We explored the possibilities of using near-infrared (NIR) spectroscopy as a prognostic biomarker for BCR-free survival (BCR-FS). Methods: Tissue specimens from 82 prostate cancer patients were obtained. Formalin-fixed paraffin-embedded slides (hematoxylin–eosin-stained) were analyzed using NIR spectroscopy. Prognostic features for BCR-FS were determined following normalization of the spectra. Results: Several differences were found throughout the NIR spectrum for the patients with or without BCR, for both the first derivative and second derivative of the NIR spectrum. Following categorization and Cox regression analysis, spectral regions at 5236 cm^−1^ (first derivative; median BCR-FS not reached versus 3.2 years; HR_high_ = 0.18 [0.08–0.39]; and *p <* 0.0001) and at 5956 cm^−1^ (second derivative; median BCR-FS not reached versus 3.8 years; HR_low_ = 0.22 [0.10–0.48]; and *p* = 0.0002) showed prognostic properties for BCR-FS. The combination of both parameters further increased the prognostic value of NIR (*p <* 0.0001). Conclusions: We demonstrated NIR spectral variations between patients with or without BCR, which have been shown to have prognostic value. This easy-to-use technique could possibly further improve post-primary radical prostatectomy monitoring and swift referral to adjuvant local therapies. Further elaboration is highly recommended to fully elucidate these variations and to gain a deeper insight into the changing chemical and physical compositions of the prostate tumor architecture.

## 1. Introduction

Prostate cancer (PCa) is the second most frequently occurring cancer, with over 1,400,000 new cases yearly, and the fifth leading cancer-related death, with 375,000 annual deaths, in men worldwide [1]. In Europe, PCa remains the most frequent cancer diagnosed, and the third leading cause of cancer deaths [2]. 

Several clinical examinations and tools, including serum prostate-specific antigen (sPSA) digital rectal examination, transrectal ultrasound, multiparametric magnetic resonance imaging and histopathological evaluation, are available to aid urologists in making a diagnosis of PCa [3,4]. Once diagnosed, three options can be offered to the patients for the treatment of local/localized PCa, depending on their status: active surveillance, radiotherapy and radical prostatectomy [3]. Radical prostatectomy encompasses the removal of the prostate and seminal vesicles, possibly combined with a pelvic lymph node dissection (PLND) [5,6]. Adjuvant radiotherapy (RT) and androgen deprivation therapy (ADT) can be added in a select patient population to reduce the risk of micrometastatic disease [7,8].

Following radical prostatectomy, PCa recurrence occurs in 27% to 53% of patients. This usually manifests as a rising sPSA concentration, which will trigger salvage therapy [9]. Although a cut-off of 0.4 ng/mL is proposed in the literature [10], the EAU-EANM-ESTRO-ESUR-SIOG guidelines suggest that salvage therapy be offered earlier to men with two consecutive sPSA rises [9]. EAU risk groups for biochemical recurrence (BCR) have been established in order to predict the risk of BCR after local treatment. This classification is based on the T stage, N stage, pre-operative sPSA concentration and International Society of Urological Pathology (ISUP) grading. Other features, such as positive surgical margins, may also be a risk factor for BCR [3,11]. Nevertheless, this EAU risk stratification is currently not used to guide the management of patients post-prostatectomy. Moreover, only a limited number of expensive tissue-based genomic predictive and prognostic biomarkers (e.g., Decipher^®^ [GenomeDx Biosciences, San Diego, CA, USA]) have emerged for disease management following BCR on prostatectomy [12,13]. It therefore remains vital to indicate which patients will have a prolonged BCR-free survival (BCR-FS) in order to optimize the post-operative management of PCa.

The use of infrared spectroscopy could be a valid asset in this field. Near-infrared (NIR) spectroscopy can provide complete information on the chemical and physical compositions of biological samples by studying vibrational transitions of tissue structures. This is accomplished by irradiating the biological samples with NIR light. This NIR light is absorbed by the molecules, resulting in a higher vibrational state that can be evaluated [14]. We have previously demonstrated that the initial diagnosis of PCa could be improved by the use of this technique [15]. Next, it was hypothesized that changes in the NIR spectrum might also have prognostic properties. The objective of the study described here is therefore to determine the prognostic effect of the NIR spectrum of radical prostatectomy specimens on BCR-FS outcome.

## 2. Materials and Methods

### 2.1. Patients and PCa Specimens

A total of 100 randomly selected prostatectomy specimens were acquired from our biobank. All surgical procedures to obtain the prostate tissues occurred between August 2014 and March 2017. Tissues without histologically proven PCa (meaning, no tumor presence in the biobank specimen; n = 4) were excluded, as well as tissues from patients presenting with metastatic disease (n = 10), who underwent prostatectomy for local recurrence (n = 2) or without documented follow-up (n = 2). The STARD flow diagram is given in Supplementary Figure S1. Hematoxylin–eosin-stained formalin-fixed paraffin-embedded (FFPE) slides (2 µm) were processed from the prostatectomy specimens, and the presence of PCa as well as the ISUP grade were histologically confirmed by a dedicated pathologist. All patient data were retrieved from the electronic patient files. Patients were followed-up in accordance to the EAU guidelines. In short, the sPSA concentration was measured every 3 to 6 months until the occurrence of BCR. BCR was defined as two consecutive rises in sPSA concentration above 0.2 µg/L [9] or early referral for salvage therapy following a single rise in sPSA concentration (whatever occurred first). The local ethics committee approved the study (13 September 2018; Belgian registration number: B670201214356) and all patients signed informed consent forms.

### 2.2. Tissue NIR Spectroscopic Analysis

NIR spectroscopy was performed in an adapted form of the method used by De Bruyne S. et al. [15,16,17]. In short, a single tissue section (normal slide) was analyzed at room temperature by means of an extended InGaAs-array-technology-enhanced NIR spectrometer (AvaSpecNIR256-2.5-HSC, Avantes, Apeldoorn, The Netherlands). Immersion oil was added onto the slides in order to eliminate the loss of resolution due to different refractive surfaces (glass versus air). Using an immobilized 50 mm integrating sphere (AvaSphere-50-LS-HAL-6-S1, Avantes), spectra of light reflections were measured across the range of 10,000 to 4000 cm^−1^. NIR spectroscopy was focused on the largest tumor area present on the section slide.

Subsequent data analysis was performed via SIMCA software version 15.0 (MKS Data Analytics Solutions). Sample preprocessing via SNV, derivatization and SG smoothing was carried out in order to remove irrelevant light scattering and to standardize the obtained NIR spectra. The proposed sample processing methods work as follows: SNV eliminates both baseline offset variations and multiplicative scaling effects, thus resulting in the accentuation of structural differences without the interference of baseline effects, such as differences in sample density and sample-to-sample measurement variations. Next, the application of spectrum derivatives enhances the resolution of the obtained spectra, which can allow for differentiation between overlapping bands. Two derivatives can be applied: the first derivative in which the rate of change of absorbance (A) with respect to wavelength (λ) is examined (dA/dλ), and the second derivative which measures alterations in the rate of change of absorbance (d^2^A/dλ^2^). Finally, enhancement of the signal-to-noise ratio without loss of spectral details can be obtained via SG smoothing. Following sample preprocessing, the prognostic properties of NIR spectroscopy on prostate tissue sections were evaluated.

### 2.3. Statistical Analysis

Statistical analyses were performed with MedCalc Statistical Software version 20.011 (MedCalc Software, Ostend, Belgium). Differences in NIR spectra, for the 1st and 2nd derivatives, between patients with and without BCR were analyzed by means of the Mann–Whitney U-test. Missing values were not included in the analyses. Intensities of significant wavenumbers were further dichotomized for survival analysis by applying the associated criterion for the highest Youden’s index (Supplementary Table S1). In short, the value with the highest Youden’s index (= sensitivity + specificity − 1) for BCR was selected as the cut-off value to discriminate between low and high difference in intensity for the respective wavenumbers. Wavenumbers, via the selected cut-off values, were then further evaluated for their prognostic properties by determination of the hazard ratio (HR) for BCR-FS by means of a 2-sided logrank (Mantel-Cox) test. BCR-FS, after up to 7 years of follow-up, was determined as time from prostatectomy until the occurrence of BCR. Patients that were lost to the follow-up were censored in the survival analysis. Survival curves were plotted using the Kaplan–Meier method. As some of the differences in intensity for the wavenumbers of interest might be associated with others, the covariate effect of prognostic wavenumbers (reaching a *p*-value < 0.05 on a univariate test) was determined via a Cox proportional hazard model for the 1st and 2nd derivatives separately, in order to determine the independent prognosticator for each derivative. The wavenumbers that were proven to be significant in this model were then used in further analysis via the creation of an ad hoc NIR combination marker. This NIR combination marker was assessed for its prognostic potential using a 2-sided logrank (Mantel-Cox) test. Finally, a multivariate analysis of this NIR combination marker and the significant clinical parameters for BCR-FS was performed using a Cox proportional hazard model. *p*-values < 0.05 were considered statistically significant.

## 3. Results

### 3.1. Patient Characteristics

Patient characteristics are given in Table 1. The majority of patients presented with ISUP grading group 2 (39%), T3 tumors (59%) and no lymph node invasion (82%). Median sPSA concentration at initial diagnosis was 8.9 ng/mL, ranging between 2.5 ng/mL and 136.4 ng/mL. Concomitant PLND was performed in 72% of patients. Next, 17% of patients received adjuvant RT, 65% of them with addition of ADT for the treatment of their N1 disease (in 80% of patients with N1 disease). Positive surgical margins were observed in 38% of cases. Distribution among EAU risk groups was 54%, 28% and 18%, for intermediate, high and very high risk groups, respectively. Twenty-eight patients (34%) presented with BCR. Having BCR was associated with the presence of positive surgical margins on the prostate specimen (*p* = 0.0022). Following prostatectomy, the median time of follow-up was 4.3 years.

### 3.2. Differences in NIR Spectra

**Figure 1:**
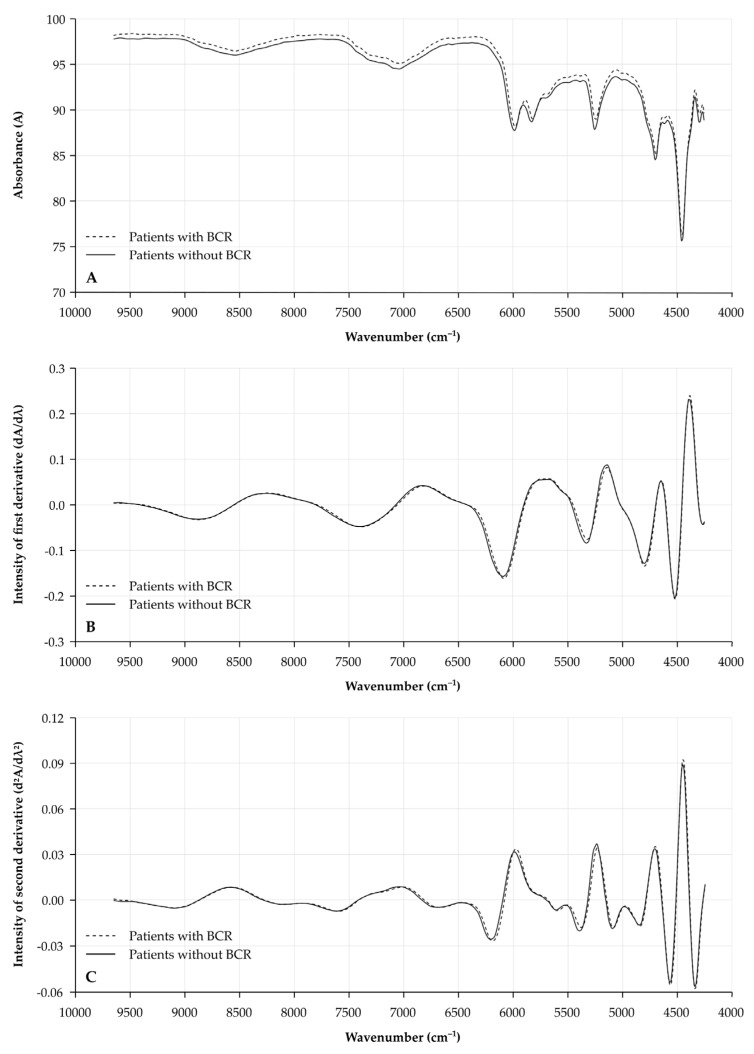
Median NIR spectra for prostatectomy specimens from patients with or without BCR. X-axis depicts wavenumber (cm^−1^). Spectra are illustrated for (**A**) the original spectra (raw data); (**B**) the 1st derivative (dA/dλ); and (**C**) the 2nd derivative (d^2^A/dλ^2^) for tissue specimens of patients with BCR (dashed line) and without BCR (full line). **A**: absorbance; λ: wavelength; and NIR: near-infrared.

Median NIR spectra for patients with and without BCR are illustrated in Figure 1. A clear shift in the original spectrum is noticed, indicating the need for normalization of the spectrum.

**Figure 2:**
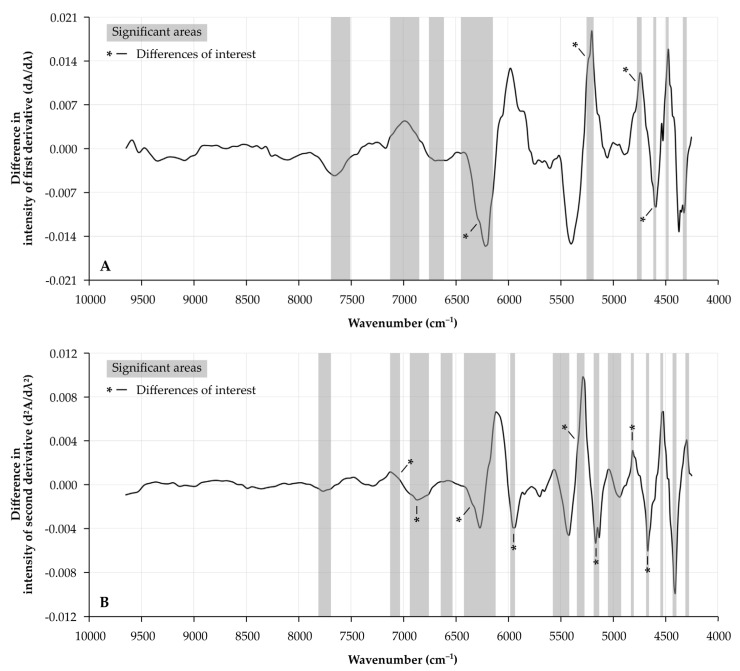
Median differences in NIR spectra of prostatectomy specimens from patients with or without BCR. X-axis depicts wavenumber (cm^−1^). Spectral differences in intensity are illustrated for (**A**) the 1st derivative (dA/dλ) and (**B**) the 2nd derivative (d^2^A/dλ^2^). Significantly different areas have been highlighted in grey. Changes reaching a significance of *p <* 0.001 have been indicated with an asterisk, and the spectral variation of these wavenumbers has been further illustrated in Supplementary Figures S2 and S3. **A**: absorbance; BCR: biochemical recurrence; λ: wavelength; and NIR: near-infrared.

Analysis of the first derivate (dA/dλ) shows several areas of interest which proved to be different between prostate specimens of patients with and without BCR (Figure 2A). Wavenumbers of interest in these areas, with significantly different intensities between patients with and without BCR, are located at 6293 cm^−1^ (*p* = 0.0002), 5236 cm^−1^ (*p* = 0.0001), 4755 cm^−1^ (*p* = 0.0004) and 4602 cm^−1^ (*p <* 0.0001).

Comparably, several differences between patients with and without BCR were observed for the second derivate (d^2^A/dλ^2^) of the NIR spectra (Figure 2B). Most significant differences were located at 7067 cm^−1^ (*p* = 0.0002), 6878 cm^−1^ (*p* = 0.0003), 6345 cm^−1^ (*p <* 0.0001), 5956 cm^−1^ (*p* = 0.0002), 5345 cm^−1^ (*p* = 0.0004), 5165 cm^−1^ (*p* = 0.0003), 4815 cm^−1^ (*p* = 0.0004) and 4671 cm^−1^ (*p* = 0.0001). All box plots for the differences in the first and second derivatives between patients with and without BCR for the above-mentioned wavenumbers are illustrated in Supplementary Figures S2 and S3. Some of these wavenumbers showed a trend towards a significant correlation with the sPSA concentrations of the patients (Supplementary Table S2).

### 3.3. BCR-FS

**Figure 3:**
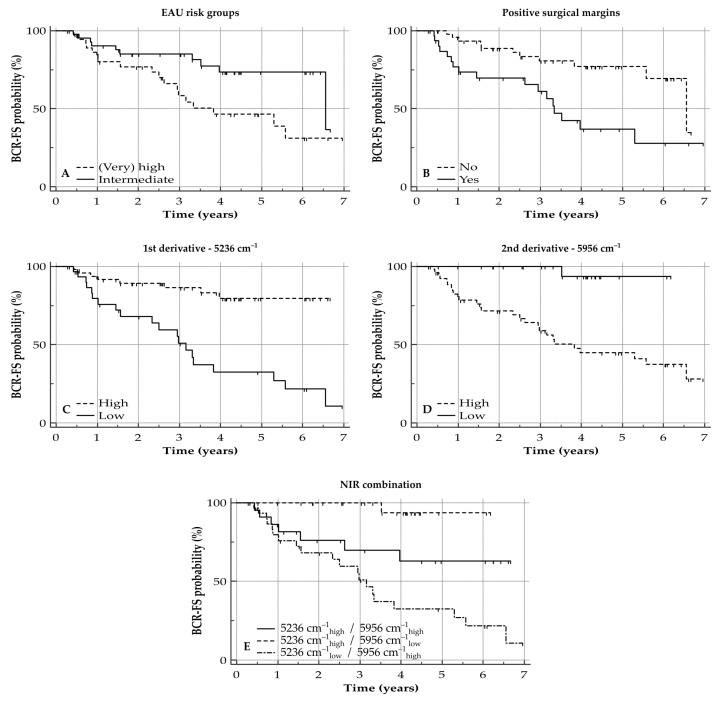
BCR-FS curves for clinical parameters and NIR spectral variations of interest. Y-axis depicts cumulative BCR-FS (%) and X-axis depicts survival time in years. BCR-FS curves are illustrated for (**A**) EAU risk groups (HR_(very) high_ = 2.42 [1.14–5.13], *p* = 0.0209); (**B**) positive surgical margins (HR_yes_ = 3.38 [1.54–7.43], *p* = 0.0024); (**C**) difference in intensity of 1st derivative at 5236 cm^−1^ (HR_high_ = 0.18 [0.08–0.39], *p <* 0.0001); (**D**) difference in intensity of 2nd derivative at 5956 cm^−1^ (HR_high_ = 4.53 [2.06–9.92], *p* = 0.0002); and (**E**) NIR combination marker (HR_5236__cm_^−1^_high/5956_ _cm_^−1^_high_ = 0.11 [0.05–0.28], HR_5236__cm_^−1^_low/5956_ _cm_^−1^_high_ = 2.52 [0.99–6.42], *p <* 0.0001). BCR-FS: biochemical recurrence-free survival; HR: hazard ratio; and NIR: near-infrared.

Univariate survival analysis of clinical parameters indicates that having a low T stage and low ISUP grading score (1–2) are associated with prolonged BCR-FS. A pathological N stage did not have any influence on BCR-FS outcome following prostatectomy, nor did sPSA concentration at initial diagnosis (according to sPSA as a tricategorical variable). The combination of the clinical parameters in the EAU risk groups showed that patients with a lower risk for BCR indeed presented with the longest BCR-FS. In addition, having positive surgical margins was associated with an early risk for BCR. All BCR-FS curves are shown in Figure 3A,B and Supplementary Figure S4; numbers are listed in Supplementary Table S3.

All wavenumbers of interest, for which the intensities that were identified as significantly different between patients with and without BCR (see Figure 1), were dichotomized and univariately assessed for their prognostic properties in BCR-FS. All proved to be significantly associated with BCR-FS. An overview is given in Supplementary Figures S5 and S6 in addition to Supplementary Table S3.

Multivariate analysis for the first and second derivatives, separately, illustrated that the difference in intensity at wavenumbers 5236 cm^−1^ for the first derivative (median BCR-FS not reached vs. 3.2 years; HR_high_ = 0.18 [0.08–0.39]; and *p <* 0.0001) and at 5956 cm^−1^ for the second derivative (median BCR-FS not reached vs. 3.8 years; HR_low_ = 0.22 [0.10–0.48]; and *p* = 0.0002) are independent prognosticators for BCR-FS following prostatectomy (Figure 3C,D; Table 2).

Combination of both wavenumbers resulted in a tricategorical prognostic biomarker which enabled prognostications for patients following prostatectomy (HR _5236 cm_^−1^ _high/5956 cm_^−1^_low_ = 0.11 [0.05–0.28], HR_5236 cm_^−1^ _low/5956 cm_^−1^_high_ = 2.52 [0.99–6.42] and *p <* 0.0001; Figure 3E). Multivariate analysis of the combination marker, next to the clinical parameters (presence of positive surgical margins and EAU risk groups), proved that the combination marker gives an added value to the presence of positive surgical margins for the BCR-FS model while the EAU risk groups are not withheld in this model (Table 2).

Next, it was determined if the newly defined NIR BCR risk groups (both the separate wavenumbers of interest as well as the NIR combination biomarker) are associated with the clinical parameters of the patients in our cohort. A significant association was observed for all NIR biomarkers with the ISUP grading groups and the pT stage. On the other hand, no association was found with the sPSA concentration nor with the N stage or choice of therapy. Subsequently, a significant association was found between the NIR biomarkers and EAU risk groups. The presence of positive surgical margins after prostatectomy was associated with the intensity at wavenumber 5236 cm^−1^ (first derivative), but not for wavenumber 5956 cm^−1^ (second derivative) or the NIR combination biomarker. All numbers are given in Supplementary Table S4.

Lastly, as the NIR biomarkers were associated with the pT stage, it was determined if the prognostic value of the NIR biomarkers was for low-volume (pT2) or high-volume disease (pT3–4). Results from this subgroup analysis were comparable for the overall population as the intensity at 5236 cm^−1^ (first derivative; *p* = 0.0001 and *p* = 0.0236), the intensity at 5956 cm^−1^ (second derivative; *p* = 0.0844 and *p* = 0.0004) and the NIR combination biomarker (*p* = 0.0004 and *p* = 0.0094) showed prognostic properties for BCR-FS in the pT2 and pT3–4 groups, respectively (Supplementary Figure S7).

## 4. Discussion

In this research we determined the prognostic features of the NIR spectrum by analyzing PCa tissue samples, obtained after primary radical prostatectomy, using NIR spectroscopy.

The patient population is well-balanced and is in line with data reported in the literature. As expected, the majority of patients presented with ISUP grading 2 or higher, T3 tumors and only locally advanced disease, as this patient population is more likely to undergo radical prostatectomy as their treatment option [3]. Positive surgical margins were found in 38% of the prostatectomy specimens. This corresponds to numbers reported in the literature on the presence of positive surgical margins in up to 50% of radical prostatectomy specimens. The high number of positive surgical margins can be attributed to the fact that approximately 60% of the tumors in our study were classified as pT3 tumors, which are associated with a higher risk for the presence of positive surgical margins [18,19]. All these clinical parameters were associated with BCR-FS outcomes. As expected, low-risk PCa—namely having low ISUP grading and a low T stage—was linked to a prolonged BCR-FS. Interestingly, sPSA concentration at initial diagnosis was not useful as a biomarker for BCR-FS after radical prostatectomy, although it was stated by Van den Broeck et al. [11] that initial sPSA concentration is an important preoperative predictor for BCR. This would indicate that initial sPSA concentration is of interest for predicting BCR, but does not provide any information on the duration of the BCR-FS. Comparably, the N stage did not exert any influence on the occurrence of BCR or on the time until BCR. This is contradictory to the literature, as lymph node metastasis is considered a worse prognostic feature for BCR-FS after radical prostatectomy [20], but can be explained by the fact that 80% of patients with N1 disease received adjuvant whole-pelvis RT, of whom 65% received adjuvant ADT.

Despite all these clinical parameters, no easy-to-use prognostic biomarker for BCR-FS has been elaborated on that can aid urologists to improve the management of PCa in the post-primary radical prostatectomy setting. Nowadays, NIR spectroscopy is a rapidly advancing, nondestructive and label-free analytical technique with high potential for clinical implementation [21]. Various research groups have applied IR spectroscopy for the diagnosis of PCa and differentiation between indolent and more aggressive PCa [15,22,23,24,25]. Recently, there has been a growing interest in prostate-specific membrane antigen-targeted NIR imaging agents to guide urologists during radical prostatectomy [26,27]. However, to our knowledge, little is known about the prognostic features of NIR spectroscopy on hematoxylin–eosin-stained FFPE slides of primary radical prostatectomy specimens in terms of BCR-FS. Focusing on the original spectra in our study, it is clear that a shift has occurred between the median absorbance for specimens of patients with and without BCR. As we cannot predict if this shift is inherent to the difference between the eventual occurrence of BCR, or is due to pre-analytical conditions (e.g., baseline offset variations, multiplicative scaling effects, differences in sample density and sample-to-sample measurement variations), only the processed spectra were considered for further BCR-FS analysis.

Comparison between the first derivative of the spectra for PCa specimens of patients with and without BCR revealed several bands with spectral variation. The most significant bands (n = 4) were located at wavenumbers 6293 cm^−1^, 5236 cm^−1^, 4755 cm^−1^ and 4602 cm^−1^. The bands at 6293 cm^−1^, 5236 cm^−1^ and 4755 cm^−1^ can be assigned to first overtone stretching of O–H bonds, second overtone stretching of C=O bonds and first overtone stretching from a carboxyl group (C−H bonds plus C=O bonds) [28,29]. These spectral variations might be attributed to carbohydrates, as O–H bonds, C=O bonds and C−H bonds are typically present in several types of monosaccharide structures, such as fucose, galactose and N-acetylglucosamines. This hypothesis is strengthened by the fact that Khajehpour et al. [30] have indicated that the NIR spectra can allow for the determination of changes in the protein fucosylation ratio. This can also be the case here, as we noticed that a high vibrational state at 5236 cm^−1^ is associated with an improved prognosis. This can be associated with the fact that a higher amount of fucosylated triantennary structures, as indicated in previous research from our group, is also associated with a prolonged BCR-FS [31]. Furthermore, the comparison for the second derivative of the NIR spectra between patients with and without BCR also revealed several spectral bands of interest (n = 8), of which most can be linked to changes observed in glycans. With regard to the variations possibly attributed to changes in glycan structures, these bands were detected at 7067 cm^−1^ (double C–H stretch [–CH2–] and C–H deformation), 6878 cm^−1^ and 6345 cm^−1^ (first overtone stretching hydrogen-bonded O–H group), 5956 cm^−1^ (first overtone C–H stretching), 5345 cm^−1^ and 5165 cm^−1^ (second overtone C=O stretching) and 4815 cm^−1^ (first overtone C=O stretching) [28,29,32]. Here, in contrast to the observation at 5236 cm^−1^, a high vibrational state at 5956 cm^−1^ was associated with a poor prognosis. This association may be explained by the fact that the presence of bisecting N-acetylglucosamines is also linked to a shorter BCR-FS [31]. It is therefore safe to assume that the NIR spectra can detect changes in the glycoprotein content in prostatectomy samples, possibly due to malignant transformation in the PCa specimens.

This apparent association between the NIR spectrum and these N-glycan patterns could explain why the NIR spectrum also plays a role as a prognosticator in BCR-FS, especially for the bands at 5236 cm^−1^ (first derivative, linked to second overtone stretching of C=O bonds) and at 5956 cm^−1^ (second derivative, linked to first overtone C–H stretching). The combination of these two spectral regions into one NIR combination marker proved to be an independent prognosticator for BCR-FS on multivariate analysis, especially as this biomarker focuses on the changes in two types of N-glycan configurations, next to the presence of positive surgical margins on the prostatectomy specimen. Using NIR spectroscopy could therefore be an asset for determining the prognosis of this patient population and to guide patient management, as the 5-year BCR-FS rate for the combination 5236 cm^−1^ _high_/5956 cm^−1^ _low_ was 94% (no BCR noticed in the first three years after prostatectomy), while the 5-year BCR-FS rate was 63% and 33% for 5236 cm^−1^ _high_/5956 cm^−1^ _high_ and 5236 cm^−1^ _low_/5956 cm^−1^ _high_, respectively. This would indicate that the latter two groups of patients will be more at risk for having BCR, and increased surveillance for these patients or earlier referral for salvage therapy might be advised.

Nevertheless, caution is advised when drawing this conclusion as it has been reported that the spectral variation noticed at 5956 cm^−1^ might also be attributed to the stretching of a cis R1–CH=CH–R2 bond. Moreover, the spectral variations seen at 4671 cm^−1^ and 4602 cm^−1^ are related to an asymmetrical stretching of–HC=CH–(=C–H stretch plus C=C stretch) [29]. These types of chemical bonds are not seen in complex N-linked carbohydrates, and it remains to be determined which compound in the prostatectomy specimens is responsible for the spectral variation seen at these wavenumbers. One possibility is that changes observed in the NIR spectrum might be associated with chemical bonds typically seen in androgens, e.g., testosterone, which are known to have a different tissue hormone exposure in prostatectomy samples and are also linked to PCa progression [33,34]. The latter would mean that the low vibrational state at 5956 cm^−1^, due to reduced androgen levels in the PCa tissue, are also associated with a poor prognosis and further progression of PCa. This hypothesis is plausible, as a trend toward significant correlation was observed between the spectral intensities and sPSA concentrations, indicating that multiple factors contribute to the observed variations in the NIR spectrum. In addition, significant associations were also found between the difference in spectral intensities and other clinical parameters, such as the ISUP grade and the pT stage. These results would indicate that in a growing and developing tumor, the chemical (and associated physical) composition is undergoing various and continuous changes. This was also demonstrated by the multivariate analysis, which indicated that one has to be mindful not only of the physical changes but also the changes in the biochemical composition of the tumor, especially in light of the prognosis and development of prostate cancer (e.g., hormone-sensitive versus castration-refractory). Moreover, the NIR biomarkers remained prognostically significant when patients were stratified according to pT stage (low-volume versus high-volume). Lastly, NIR spectroscopy can also visualize changes observed in O-glycosylation, which may be present in prostate tissue, or reduced water content in PCa cells [35].

Therefore, further elaboration is needed to fully elucidate the NIR spectral variations seen in prostatectomy specimens, thus resulting in a deeper insight into the changing chemical and physical compositions of the tumor architecture.

Despite the interesting findings reported, our research also faces some limitations. Firstly, the prostate gland is a very heterogeneous organ. It may therefore be assumed that only limited changes or a part of the changes can be observed by focusing on a small fraction of the prostate specimen derived through radical prostatectomy. In addition, both normal tissue and PCa tissue can be present on the same tissue slide. As a result, NIR spectroscopy might also analyze normal tissue adjacent to the tumor material present on the tissue slide. By explicitly focusing on the largest tumor area present on the section slide, we have attempted to minimize this issue. Nevertheless, it remains reasonable that normal tissue was also analyzed next to the PCa tissue from each patient. The fact that, even in this setting, highly significant changes in the NIR spectrum could be observed between patients with and without BCR would indicate that chemical changes are already induced in other (healthy/normal) prostate tissue prior to the cellular changes which can be observed via microscopy. In this way, NIR spectroscopy shows an added value to the currently used techniques for follow-up in PCa. Nonetheless, it remains to be seen if the outcome in the NIR spectrum of serially analyzed tissue slides is consistent or not. Future evaluation of serial samples, taken from the same prostatectomy sample (intra-tumor) and over time, is therefore advised to determine the robustness of the NIR spectrum for heterogeneous or damaged tissue sections [36]. Secondly, the number of PCa tissue samples derived from primary radical prostatectomy (n = 82) in our study is relatively small in comparison to the number of radical prostatectomies that take place yearly. Even though the number of patients with BCR was in line with the number reported in the literature (34%), this number does not allow for deeper multivariate statistical analysis, for which a larger cohort will be needed. This is unfortunate in light of the recently adapted EAU-EANM-ESTRO-ESUR-SIOG guidelines, in which the idea was stipulated for a new risk stratification that combined ISUP grading with PSA doubling time. Based on the risk, patients with BCR will have a shorter or prolonged metastasis-free survival [9,37]. It has been proven that reorganization of the extracellular matrix, a process also driven by glycoproteins, can lead to an escape of PCa cells from the prostate gland, with consequent local or distant metastatic disease [38,39]. As these changes in the chemical and physical compositions of the extracellular matrix might also be visualized via NIR spectroscopy, it would be worthwhile to investigate the effect of changes in the NIR spectrum and its prognostic properties on metastasis-free survival next to the newly proposed risk stratification.

## 5. Conclusions

In this retrospective study, we were able to identify several regions in the NIR spectrum of radical prostatectomy specimens for which variations existed between patients with and without BCR. From the identified bands in the NIR spectrum, changes in the first derivative at 5236 cm^−1^ and in the second derivative at 5956 cm^−1^ were shown to be potent prognosticators for BCR-FS. Although a possible association exists between the observed variations in the NIR spectrum and changes in the glycoproteome of malignant prostate tissue, further elaboration is needed to fully elucidate the NIR spectral variations described here, thus resulting in a deeper insight into the changing chemical and physical compositions of the prostate tumor architecture. Secondly, large-scale validation in an independent patient population is needed to determine the true prognostic potential of NIR spectroscopy following primary radical prostatectomy.

## Figures and Tables

**Table 1:** Patient characteristics.

Number of patients	82 (100)
Age at initial diagnosis, years	64 (48–78)
sPSA concentration at initial diagnosis	
<10 ng/mL	44 (53)
10 ng/mL–20 ng/mL	26 (32)
>20 ng/mL	12 (15)
ISUP grading group	
1	6 (7)
2	32 (39)
3	16 (20)
4	11 (13)
5	17 (21)
pT stage	
2	33 (40)
3	48 (59)
4	1 (1)
N stage	
0	67 (82)
1	15 (18)
EAU risk group	
Intermediate	44 (54)
High	23 (28)
Very High	15 (18)
Therapy	
Radical prostatectomy	23 (28)
Prostatectomy + PLND	45 (55)
Prostatectomy + PLND + RT	5 (6)
Prostatectomy + PLND + RT + ADT	9 (11)
Surgical margins	
Positive	31 (38)
Negative	51 (62)
BCR	
Yes	28 (34)
No	54 (66)

All data are n (%) except for sPSA concentration and age at initial diagnosis: median (range). EAU risk groups, based on ISUP grading score, sPSA concentration and TNM classification, were defined as stipulated in the EAU-EANM-ESTRO-ESUR-SIOG guidelines [3]. T and N stages are based on the pathological result. If no PLND was performed, the N stage is based on the outcome of a CT/MRI scan. ADT, androgen deprivation therapy; BCR, biochemical recurrence; EAU, European Association of Urology; ISUP, International Society of Urological Pathology; PLND, pelvic lymph node dissection; RT, radiotherapy; and sPSA, serum prostate-specific antigen.

**Table 2:** Cox proportional hazard model for BCR-FS.

Parameter	Univariate		Multivariate *	
	HR (95% CI)	*p*-Value	HR (95% CI)	*p*-Value
EAU risk groups				
Intermediate	1		1	
(Very) High	2.42 (1.14–5.13)	0.0209	0.88 (0.36–2.14)	0.7789
Positive surgical margins				
No	1		1	
Yes	3.38 (1.54–7.43)	0.0024	2.84 (1.33–6.09)	0.0073
Intensity at 5236 cm^−1^ (1st derivative)				
Low	1			
High	0.18 (0.08–0.39)	<0.0001		
Intensity at 5956 cm^−1^ (2nd derivative)				
Low	1			
High	4.53 (2.06–9.92)	0.0002		
NIR combination marker (3 categories)				
5236 cm^−1^ _high_/5956 cm^−1^ _high_	1		1	
5236 cm^−1^ _high_/5956 cm^−1^ _low_	0.11 (0.05–0.28)	<0.0001	0.06 (0.01–0.46)	0.0067
5236 cm^−1^ _low_/5956 cm^−1^ _high_	2.52 (0.99–6.42)		2.02 (0.83–4.92)	0.1192

* Due to the low number of events (n = 28), only three variables were included in the multivariate Cox proportional hazard analysis: EAU risk groups, positive surgical margins and the NIR combination marker. T stage, sPSA concentration and ISUP grading score were excluded as the EAU risk groups are a combination of parameters based on TNM classification, sPSA and ISUP grading score. 95% CI: 95% confidence interval; BCR-FS: biochemical recurrence; EAU: European Association of Urology; HR: hazard ratio; ISUP: International Society of Urological Pathology; NIR: near-infrared; and sPSA: serum prostate-specific antigen.

## Data Availability

The data presented in this study are available on request from the corresponding author. The data are not publicly available due to European General Data Protection Regulation.

## Supplementary Materials

The following are available online at https://www.mdpi.com/article/10.3390/cancers13236034/s1: Figure S1: STARD flowchart diagram of PCa tissue samples included, Figure S2: Difference in intensity in NIR spectra for wavenumbers of interest (1st derivative), Figure S3: Difference in intensity in NIR spectra for wavenumbers of interest (2nd derivative), Figure S4: BCR-FS curves for clinical parameters, Figure S5: BCR-FS curves for wavenumbers of interest with difference in intensity in NIR spectra (1st derivative), Figure S6: BCR-FS curves for wavenumbers of interest with difference in intensity in NIR spectra (2nd derivative), Figure S7: BCR-FS curves for independent prognostic wavenumbers, stratified according to pT stage, Table S1: Optimal cut-off value for BCR-FS, Table S2: Correlation between NIR and sPSA concentrations, Table S3: Univariate BCR-FS outcome, Table S4: Overview of distribution of clinical parameters per defined NIR BCR risk groups.
