# Supplementary material for: Prognostic Features of Near-Infrared Spectroscopy Following Primary Radical Prostatectomy
Source: Cancers (Basel). 2021 Nov 30;13(23):6034. doi: 10.3390/cancers13236034 (PMC8656494; doi:10.3390/cancers13236034)
Supplement: Supplementary file 1 [file cancers-13-06034-s001.zip › cancers-1423205-supplementary.pdf]

Prognostic features of near infrared spectroscopy following primary radical prostatectomy

Supplementary materials

FIGURES 2

- Figure S1: STARD flowchart diagram of PCa tissue samples included 2
- Figure S2: Difference in intensity in NIR spectra for wavenumbers of interest (1st derivative) 3
- Figure S3: Difference in intensity in NIR spectra for wavenumbers of interest (2nd derivative) 4
- Figure S4: BCR-FS curves for clinical parameters 5
- Figure S5: BCR-FS curves for wavenumbers of interest with difference in intensity in NIR spectra (1st derivative) 6
- Figure S6: BCR-FS curves for wavenumbers of interest with difference in intensity in NIR spectra (2nd derivative) 7
- Figure S7**:** BCR-FS curves for independent prognostic wavenumbers, stratified according to pT stage 8

TABLES 9

- Table S1: Optimal cut-off value for BCR-FS 9
- Table S2: Correlation between NIR and sPSA concentrations 10
- Table S3: Univariate BCR-FS outcome 11
- Table S4: Overview of distribution of clinical parameters per defined NIR BCR risk groups 13

FIGURES

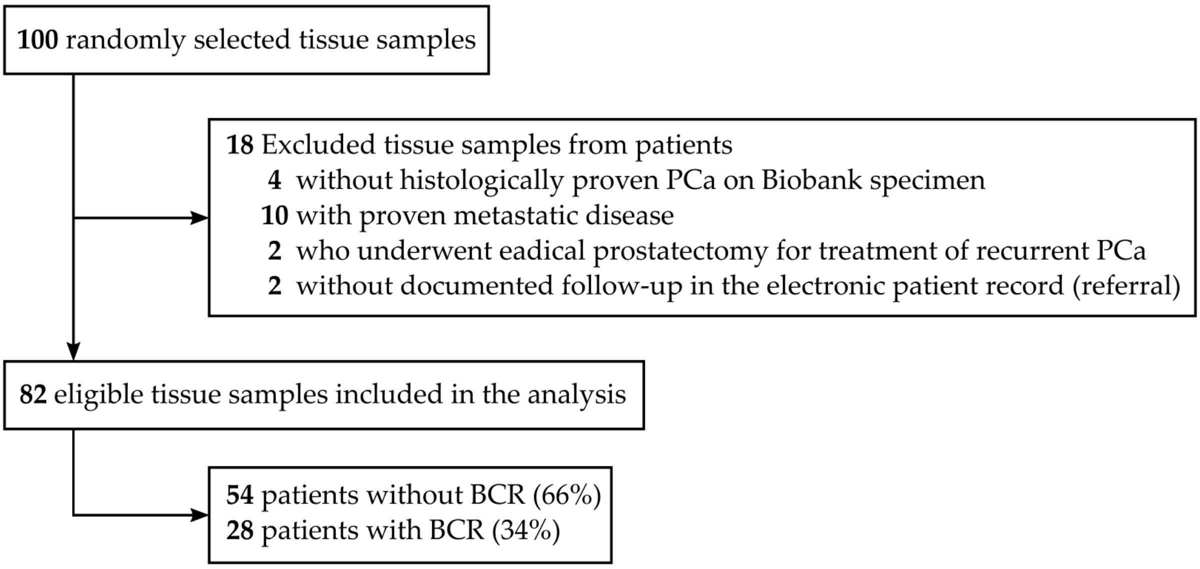

Figure S1: STARD flowchart diagram of PCa tissue samples included. BCR, biochemical recurrence; PCa, prostate cancer.

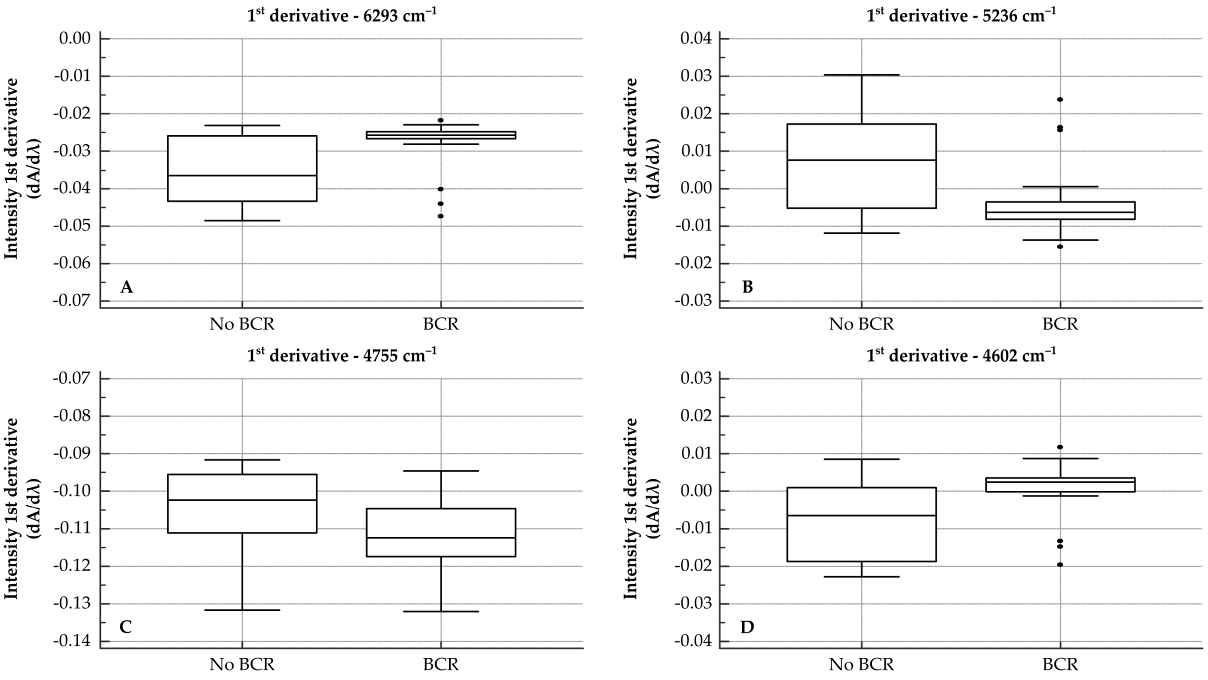

Figure S2: Difference in intensity in NIR spectra for wavenumbers of interest (1st derivative). Plots are given for the differences in the NIR spectra of prostate specimens from patients with or without BCR. Comparisons are illustrated for wavenumbers: A. 6293 cm^{-1} ($P = 0.0002$); B. 5236 cm^{-1} (P = 0. 0001); C. 4755 cm^{-1} (P = 0. 0004); and D. 4602 cm^{-1} (P < 0. 0001). BCR, biochemical recurrence; NIR, near infrared.

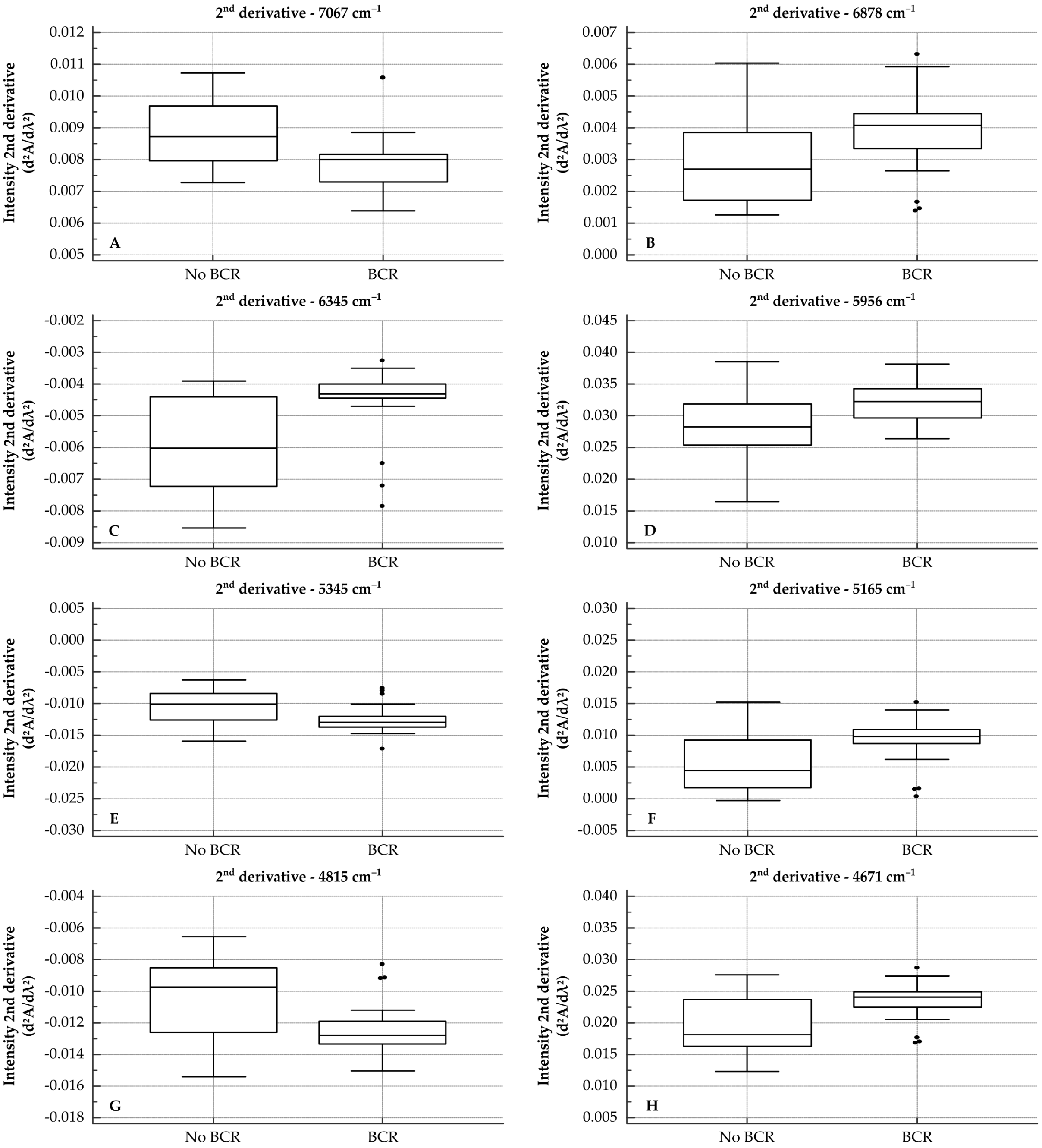

Figure S3: Difference in intensity in NIR spectra for wavenumbers of interest (2nd derivative). Plots are given for the differences in the NIR spectra of prostate specimens from patients with or without BCR. Comparisons are illustrated for wavenumbers: A. 7067 cm^{-1} (P = 0.0002); B. 6878 cm^{-1} (P = 0.0003); C. 6345 cm^{-1} (P < 0.0001); D. 5956 cm^{-1} (P = 0.0002); E. 5345 cm^{-1} (P = 0.0004); F. 5165 cm^{-1} (P = 0.0003); G. 4815 cm^{-1} (P = 0.0004); and H. 4671 cm^{-1} (P = 0.0001). BCR, biochemical recurrence; NIR, near infrared.

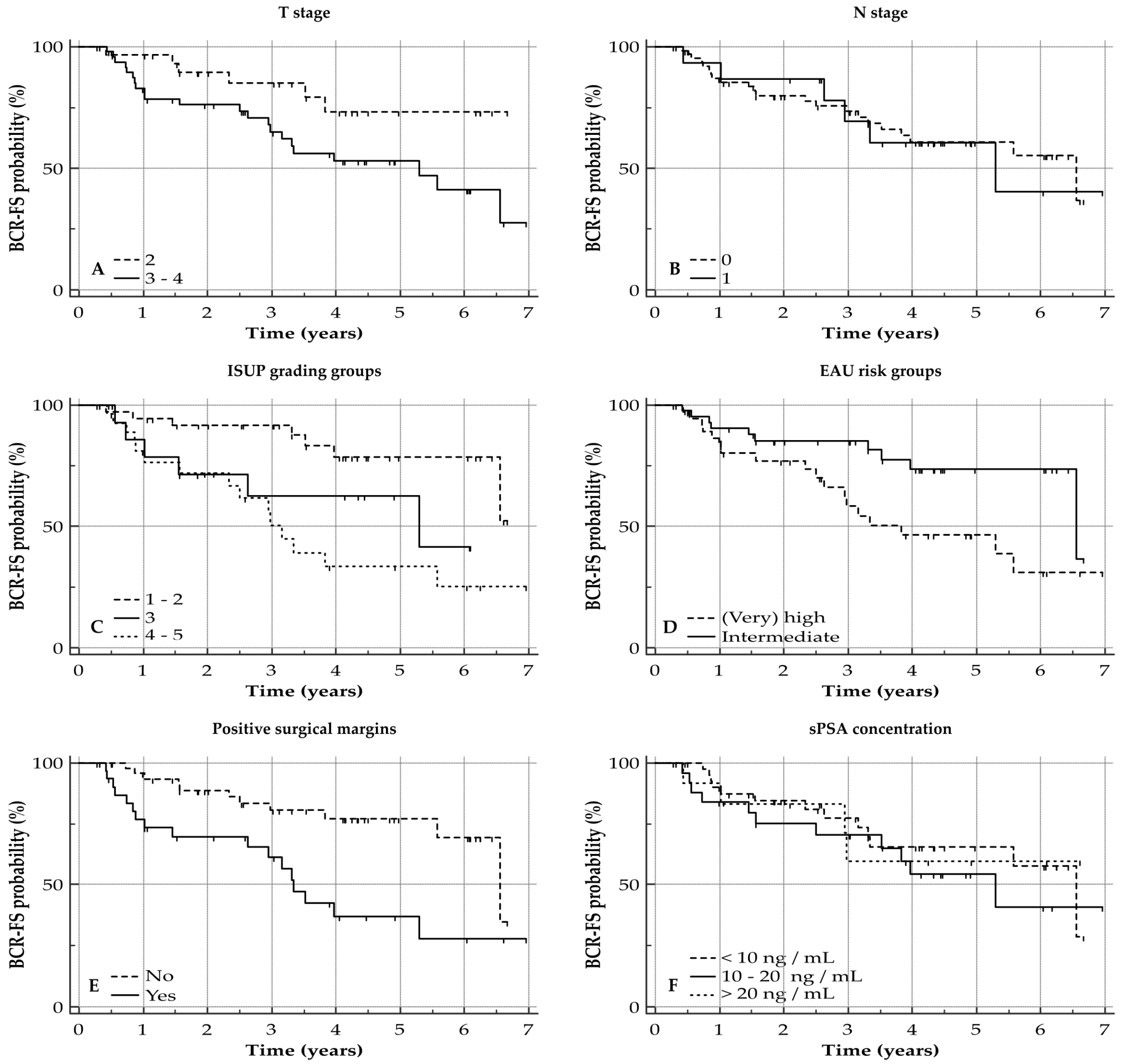

Figure S4: **BCR-FS curves for clinical parameters**. Y-axis depicts cumulative BCR-FS (%), X-axis depicts survival time in years. BCR-FS curves are illustrated for A. T stage (HR_{3-4} = 2.23 [1.04 – 4.74], P = 0.0381); B. N stage (HR_{1} = 1.06 [0.42 – 2.64], P = 0.9086); C. ISUP grading groups (HR_{3} = 2.63 [0.96 – 7.25], HR_{4-5} = 4.18 [1.77 – 9.92], P = 0.0033); D. EAU risk groups ($HR_{(very)\ high}$ = 2.42 [1.14 – 5.13], P = 0.0209); E. positive surgical margins (HR_{yes} = 3.38 [1.54 – 7.43], P = 0.0024); and F. sPSA concentration (HR_{10-20} = 1.39 [0.60 – 3.20], $HR_{>20}$ = 1.02 [0.35 – 2.96], P = 0.6998). BCR-FS, biochemical recurrence-free survival; EAU, European Association of Urology; HR, hazard ratio; sPSA, serum prostate specific antigen.

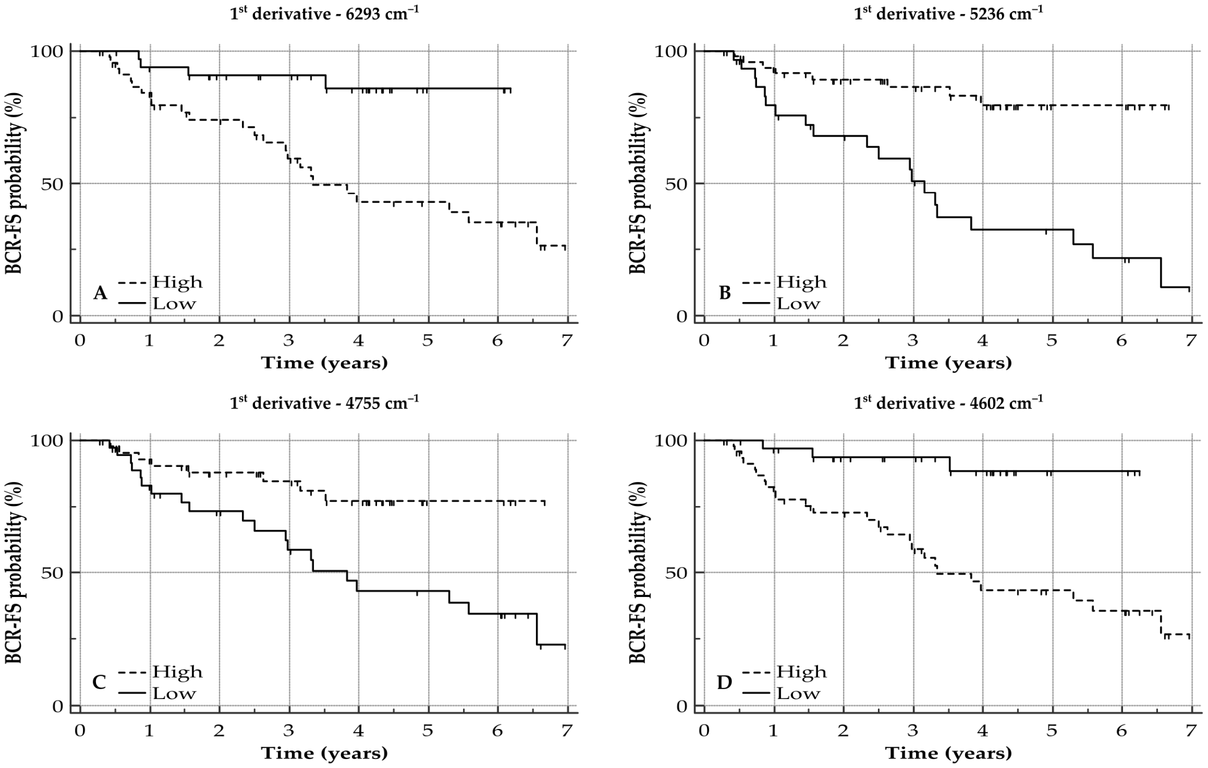

Figure S5: BCR-FS curves for wavenumbers of interest with difference in intensity in NIR spectra (1st derivative). Y-axis depicts cumulative BCR-FS (%), X-axis depicts survival time in years. BCR-FS curves are illustrated for wavenumbers: A. 6293 cm^{-1} (HR_{high} = 3.73 [1.73 – 8.01], P = 0.0008); B. 5236 cm^{-1} (HR_{high} = 0.18 [0.08 – 0.39], P < 0.0001); C. 4755 cm^{-1} (HR_{high} = 0.33 [0.15 – 0.71], P = 0.0042); and D. 4602 cm^{-1} (HR_{high} = 4.06 [1.89 – 8.74], P = 0.0003). BCR-FS, biochemical recurrence-free survival; HR, hazard ratio; NIR, near infrared.

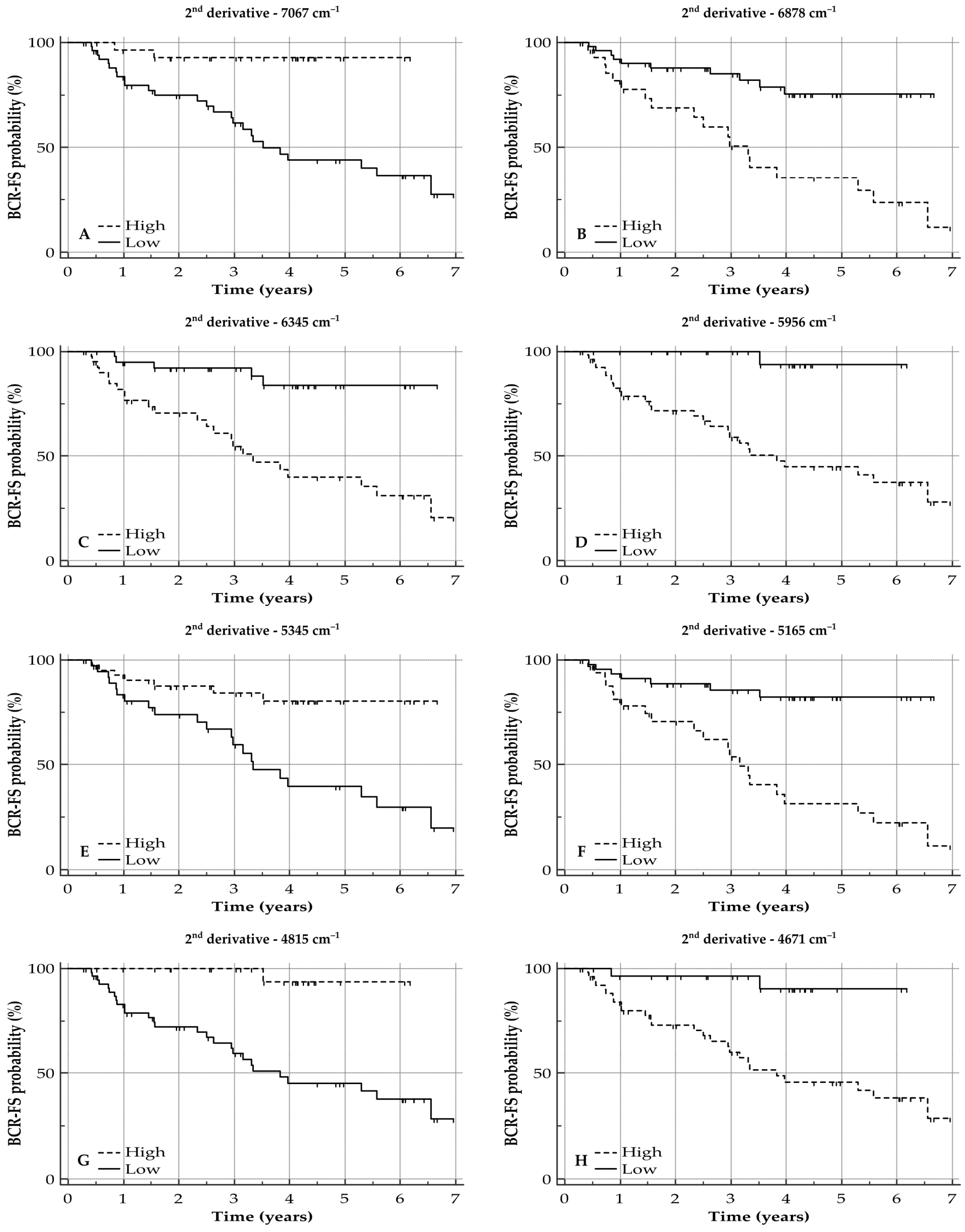

Figure S6: BCR-FS curves for wavenumbers of interest with difference in intensity in NIR spectra (2nd derivative). Y-axis depicts cumulative BCR-FS (%), X-axis depicts survival time in years. BCR-FS curves are illustrated for wavenumbers: A. 7067 cm^{-1} (HR_{high} = 0.25 [0.11 – 0.54], P = 0.0005); B. 6878 cm^{-1} (HR_{high} = 4.65 [2.08 – 10.4], P = 0.0002); C. 6345 cm^{-1} (HR_{high} = 4.38 [2.06 – 9.30], P = 0.0001); D. 5956 cm^{-1} (HR_{high} = 4.53 [2.06 – 9.92], P = 0.0002); E. 5345 cm^{-1} (HR_{high} = 0.28 [0.13 – 0.59], P = 0.0009); F. 5165 cm^{-1} (HR_{high} = 5.36 [2.47 – 11.6], P < 0.0001); G. 4815 cm^{-1} (HR_{high} = 0.23 [0.10 – 0.50], P = 0.0002); and H. 4671 cm^{-1} (HR_{high} = 3.89 [1.78 – 8.52], P = 0.0007). BCR-FS, biochemical recurrence-free survival; HR, hazard ratio; NIR, near infrared.

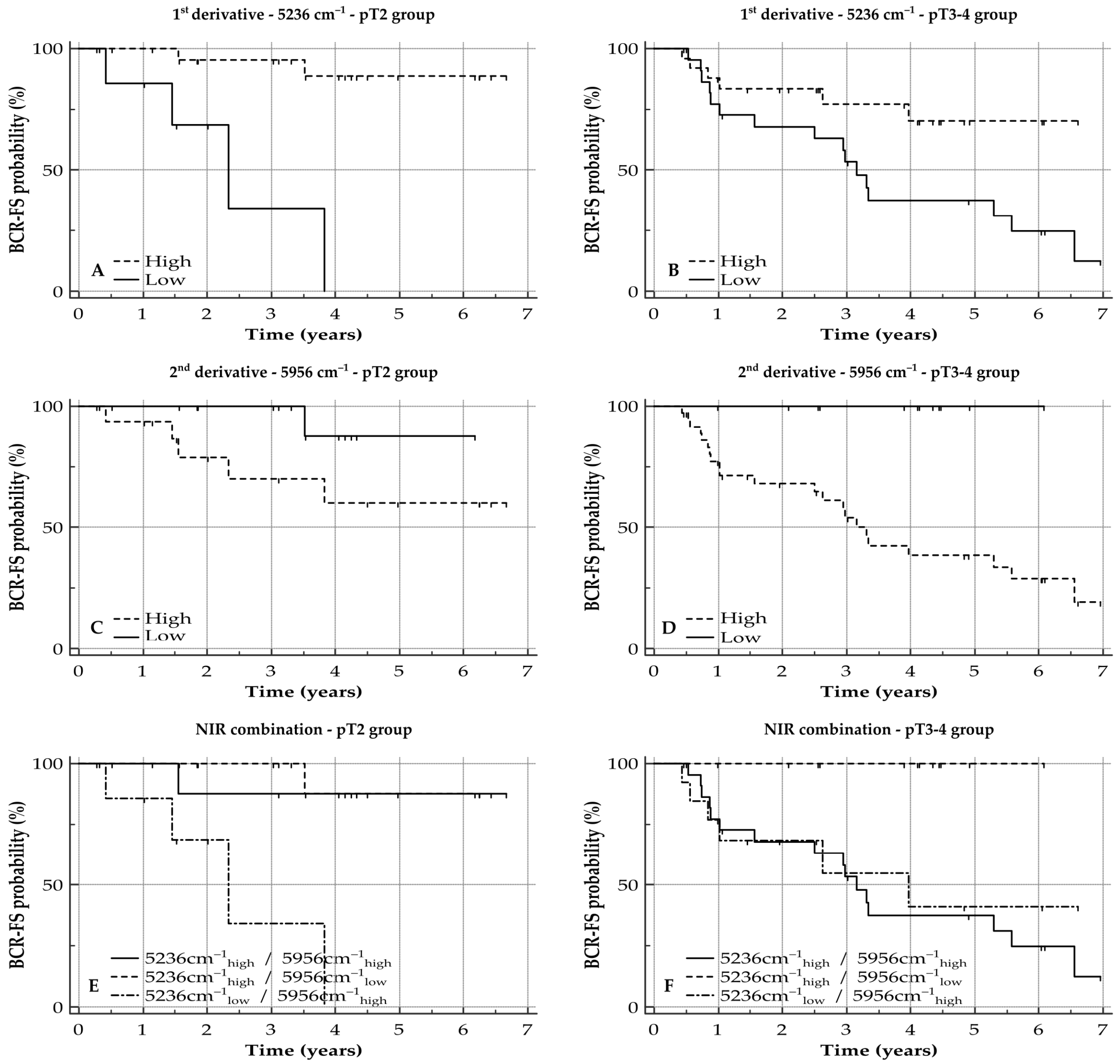

Figure S7: BCR-FS curves for independent prognostic wavenumbers, stratified according to pT stage. Y-axis depicts cumulative BCR-FS (%), X-axis depicts survival time in years. BCR-FS curves are illustrated for wavenumbers: A. 5236 cm^{-1} (1st derivative, pT2 group; P = 0.0001); B. 5236 cm^{-1} (1st derivative, pT3-4 group; P = 0.0236); C. 5956 cm^{-1} (2nd derivative, pT2 group; P = 0.0844); B. 5956 cm^{-1} (2nd derivative, pT3-4 group; P = 0.0027); E. NIR combination marker (pT2 group; P = 0.0004); and NIR combination marker (pT3-4 group; P = 0.0094). BCR-FS, biochemical recurrence-free survival; HR, hazard ratio; NIR, near infrared

TABLES

Table S1: Optimal cut-off value for BCR-FS

Derivative	Wavenumber	
1st	6293 cm^{-1}	> -0.0275322
	5236 cm^{-1}	> -0.00556171
	4755 cm^{-1}	> -0.109767
	4602 cm^{-1}	> -0.00151998
2nd	7067 cm^{-1}	> 0.00870198
	6878 cm^{-1}	> 0.00389755
	6345 cm^{-1}	> -0.00448452
	5956 cm^{-1}	> 0.027346
	5345 cm^{-1}	> -0.0122063
	5165 cm^{-1}	> 0.00922149
	4815 cm^{-1}	> -0.00913882
	4671 cm^{-1}	> 0.0174151

BCR-FS, biochemical recurrence-free survival

Table S2: Correlation between NIR and sPSA concentrations

Derivative	Wavelength	*r*	P value
1st	6293 cm^{-1}	-0.195	0.0812
	5236 cm^{-1}	0.191	0.0876
	4755 cm^{-1}	0.112	0.3215
	4602 cm^{-1}	-0.212	0.0576
2nd	7067 cm^{-1}	0.188	0.0937
	6878 cm^{-1}	-0.169	0.1325
	6345 cm^{-1}	-0.193	0.0843
	5956 cm^{-1}	-0.167	0.1367
	5345 cm^{-1}	0.157	0.1623
	5165 cm^{-1}	-0.168	0.1341
	4815 cm^{-1}	0.149	0.1847
	4671 cm^{-1}	-0.180	0.1074

Correlation were calculated using Pearson's correlation coefficient *r*. NIR, near infrared; sPSA, serum prostate-specific antigen

Table S3: Univariate BCR-FS outcome

Parameter	HR (95% CI)	P value
**sPSA concentration at initial diagnosis**		
< 10 ng/mL	1	
10 ng/mL – 20 ng/mL	1.39 (0.60 – 3.19)	0.6998
> 20 ng/mL	1.02 (0.35 – 2.96)	
**T stage**		
2	1	
3-4	2.23 (1.04 – 4.74)	0.0381
**N stage**		
0	1	
1	1.06 (0.42 – 2.64)	0.9086
**ISUP grade group**		
1 – 2	1	
3	2.63 (0.96 – 7.25)	0.0033
4 – 5	4.18 (1.77 – 9.92)	
**EAU risk groups**		
Intermediate	1	
(Very) high	2.42 (1.14 – 5.13)	0.0209
**Positive surgical margins**		
No	1	
Yes	3.38 (1.54 – 7.43)	0.0024
**Intensity at 6293 cm^{-1} (1st derivative)**		
Low	1	
High	3.73 (1.73 – 8.01)	0.0008
**Intensity at 5236 cm^{-1} (1st derivative)**		
Low	1	
High	0.18 (0.08 – 0.39)	< 0.0001
**Intensity at 4755 cm^{-1} (1st derivative)**		
Low	1	
High	0.33 (0.15 – 0.71)	0.0042
**Intensity at 4602 cm^{-1} (1st derivative)**		
Low	1	
High	4.06 (1.89 – 8.74)	0.0003
**Intensity at 7067 cm^{-1} (2nd derivative)**		
Low	1	
High	0.25 (0.11 – 0.54)	0.0005
**Intensity at 6878 cm^{-1} (2nd derivative)**		
Low	1	
High	4.65 (2.08 – 10.4)	0.0002
**Intensity at 6345 cm^{-1} (2nd derivative)**		
Low	1	
High	4.38 (2.06 – 9.30)	0.0001
**Intensity at 5956 cm^{-1} (2nd derivative)**		
Low	1	
High	4.53 (2.06 – 9.92)	0.0002
**Intensity at 5345 cm^{-1} (2nd derivative)**		
Low	1	
High	0.28 (0.13 – 0.59)	0.0009
**Intensity at 5165 cm^{-1} (2nd derivative)**		
Low	1	

High	5.36 (2.47 – 11.6)	< 0.0001
**Intensity at 4815 cm^{-1} (2nd derivative)**		
Low	1	
High	0.23 (0.10 – 0.50)	0.0002
**Intensity at 4671 cm^{-1} (2nd derivative)**		
Low	1	
High	3.89 (1.78 – 8.52)	0.0007
**NIR combination marker (3 categories)**		
5236 cm^{-1} $_{high}$ / 5956 cm^{-1} $_{high}$	1	
5236 cm^{-1} $_{high}$ / 5956 cm^{-1} $_{low}$	0.11 (0.05 – 0.28)	< 0.0001
5236 cm^{-1} $_{low}$ / 5956 cm^{-1} $_{high}$	2.52 (0.99 – 6.42)	

95% CI, 95% confidentiality interval; BCR-FS, biochemical recurrence-free survival; EAU, European Association of Urology; HR, hazard ratio; ISUP, International Society of Urological Pathology; sPSA, serum prostate specific antigen.

Table S4: Overview of distribution of clinical parameters per defined NIR BCR risk groups

Parameter		1st derivative – 5236 cm^{-1}			2nd derivative – 5956 cm^{-1}			NIR combination biomarker			
		Low	High	P	Low	High	P	5236 cm^{-1} high / 5956 cm^{-1} high	5236 cm^{-1} high / 5956 cm^{-1} low	5236 cm^{-1} low / 5956 cm^{-1} high	P
**sPSA (ng / mL)**		10.9 (2.5 - 56.1)	8.3 (3.9 - 136.4)	0.3898	8.8 (2.5 - 56.1)	11.9 (4.0 - 136.4)	0.5053	7.5 (3.9 - 44.1)	11.9 (4.0 - 136.4)	10.9 (2.5 - 56.1)	0.2672
**ISUP grading group**	1	5	1		1	5		0	5	1	
	2	25	7		21	11		14	11	7	
	3	11	5		9	7		4	7	5	
	4	5	6		9	2		3	2	6	
	5	5	12	0.0074	14	3	0.0340	2	3	12	0.0055
**pT stage**	2	26	7		17	16		10	16	7	
	3-4	25	24	0.0115	37	12	0.0255	13	12	24	0.0242
**N stage**	0	41	26		46	21		20	21	26	
	1	10	5	0.6945	8	7	0.2609	3	7	5	0.5056
**EAU risk group**	Intermediate	35	9		24	20		4	2	17	
	High	6	17		21	2		15	20	9	
	Very High	10	5	0.0001	9	6	0.0093	4	6	5	0.0007
**Therapy**	Radical P	17	6		14	9		8	9	6	
	P +PLND	25	20		32	13		12	13	20	
	P+PLND+RT	4	1		3	2		2	2	1	
	P+PLND+RT+ADT	5	4	0.3871	5	4	0.7273	1	4	4	0.6330
**Surgical margins**	Negative	36	15		31	20		16	20	15	
	Positive	15	16	0.0457	23	8	0.2172	7	8	16	0.1313

All data are n (%) except for sPSA: median (range). EAU risk groups, based on ISUP grading score, sPSA and TNM classification, were defined as stipulated in the EAU-EANM-ESTRO-ESUR-SIOG guidelines. T and N stage are based on the pathological result. If no PLND was performed, N stage is based on the outcome of the CT/MRI scan. ADT, androgen deprivation therapy; BCR, biochemical recurrence; EAU, European Association of Urology; ISUP, International Society of Urological Pathology; NIR, near infrared; P, prostatectomy; PLND, pelvic lymph node dissection; RT, radiotherapy; sPSA, serum prostate specific antigen.
